# Ectodermal dysplasia: aggressive treatment of oligodontia versus non-intervention?

**DOI:** 10.1186/1746-160X-8-S1-O3

**Published:** 2012-05-25

**Authors:** KS Kurtz

**Affiliations:** 1 New York University College of Dentistry, USA

## 

Prosthodontists can serve as productive members of the craniofacial team and can help coordinate multidisciplinary treatment of patients with congenital anomalies. Often there are few, if any, evidence-based criteria to assist them in developing “correct” clinical protocols. When syndromes involve hypodontia, oligodontia, or anodontia, coordinating prosthetic treatment through phases of three-dimensional growth and development can be daunting.

Early implant intervention with anodontia patients is acceptable in some treatment centers. Correcting previously untreated adolescent oligodontia can be extremely challenging and removal of strategic teeth with immediate implant placement is surely controversial. Understanding the morbidity of grafting in these patients and the questionable long-term survival of overdenture abutment teeth will lend to an active discussion of the advocated aggressive therapeutic approach.

